# Transcranial alternating current stimulation over multiple brain areas with non-zero phase delays other than 180 degrees modulates visuospatial working memory performance

**DOI:** 10.1038/s41598-023-39960-3

**Published:** 2023-08-05

**Authors:** Jimin Park, Sangjun Lee, Seonghun Park, Chany Lee, Sungshin Kim, Chang-Hwan Im

**Affiliations:** 1 Department of Electronic Engineering, Hanyang University, 222 Wangsimni-ro, Seongdong-gu, Seoul, 133-791 Republic of Korea; 2 Cognitive Science Research Group, Korea Brain Research Institute, Daegu, Republic of Korea; 3 Department of Cognitive Sciences, Hanyang University, Seoul, Republic of Korea; 4 Department of Biomedical Engineering, Hanyang University, Seoul, Republic of Korea

**Keywords:** Biomedical engineering, Cognitive neuroscience, Computational neuroscience

## Abstract

While zero-phase lag synchronization between multiple brain regions has been widely observed, relatively recent reports indicate that systematic phase delays between cortical regions reflect the direction of communications between cortical regions. For example, it has been suggested that a non-zero phase delay of electroencephalography (EEG) signals at the gamma frequency band between the bilateral parietal areas may reflect the direction of communication between these areas. We hypothesized that the direction of communication between distant brain areas might be modulated by multi-site transcranial alternating current stimulation (tACS) with specific phase delays other than 0° and 180°. In this study, a new noninvasive brain stimulation (NIBS) method called multi-site multi-phase tACS (msmp-tACS) was proposed. The efficacy of the proposed method was tested in a case study using a visuospatial working memory (VWM) paradigm in which the optimal stimulation conditions including amplitudes and phases of multiple scalp electrodes were determined using finite element analysis adopting phasor representation. msmp-tACS was applied over the bilateral intraparietal sulci (IPS) and showed that 80 Hz tACS with the phase for the right IPS leading that for the left IPS by 90° (= 3.125 ms) partialized VWM performance toward the right visual hemifield. The three stimulation conditions were synchronized, RL, and LR, which refers to stimulation condition with no phase lag, stimulation phase of right IPS (rIPS) leading left IPS (lIPS) by 90° and the stimulation of lIPS leading rIPS by 90°, respectively. The lateralization of VWM significantly shifted towards right visual hemifield under the RL condition compared to the synchronized and LR conditions. The shift in VWM was the result of the stimulation affecting both left and right visual hemifield trials to certain degrees, rather than significantly increasing or decreasing VWM capacity of a specific visual hemifield. Altered brain dynamics caused by msmp-tACS partialized VWM performance, likely due to modulation of effective connectivity between the rIPS and lIPS. Our results suggest that msmp-tACS is a promising NBS method that can effectively modulate cortical networks that cannot be readily modulated with conventional multi-site stimulation methods.

## Introduction

Transcranial alternating current stimulation (tACS) is a noninvasive brain stimulation (NIBS) method that allows for modulation of neuronal oscillations. It is believed that tACS entrains endogenous brain activities to the input current waveforms by applying a weak alternating current through scalp electrodes^1^. It has been demonstrated that the amplitude, frequency, and phase of the stimulation current are regarded as important factors that determine the efficacy of tACS. Indeed, the frequency-specific effects of tACS have been reported in multiple studies^2–4^, in which the stimulation frequencies were selected based on brain imaging methods including electroencephalography (EEG) and functional magnetic resonance imaging (fMRI). Additionally, recent studies determined a simulation frequency using complex EEG characteristics, such as cross-frequency coupling^5^, counteraction of EEG bands^6^, or P300 components^7^.

While most of the previous studies focused on the modulation of single brain region activities, evidence suggests that synchronization between distant cortical regions may also provide a useful information for understanding conscious perception and cognition^8–10^. These studies reported that the communication between cortical areas is observed in the form of synchronous coupling at the gamma band (30–100 Hz), which is also referred to as the communication through coherence (CTC) hypothesis^11^. In compliance with the CTC hypothesis, multi-site tACS has been introduced to modulate the synchronization between multiple brain regions. However, the reported results were confounding; tACS-induced synchronization of two cortical regions enhanced shape perception^8^ and working memory (WM)^12^, whereas auditory motor mapping^13^ and right ear advantage^14^ were unaffected. Additionally, bistable motion perception was enhanced when the parietal-occipital cortex was desynchronized by applying tACS^15^.

Traditionally, phase differences between cortical regions have not been seriously considered in previous multi-site tACS studies. Recently, some tACS studies attempted to simultaneously stimulate two cortical areas using alternating currents (ACs) with a 180° phase difference at the designated stimulation frequency in order to maximize the modulatory effect^16–20^. However, no studies have attempted to stimulate multiple cortical regions with ACs with non-zero phase delays other than 180°.

Indeed, Reinhart et al.^19^ reported the importance of synchronization of multiple cortical regions on WM performance and further suggested that tACS with a non-zero phase delay may have the potential to provide new insights to understand the roles of subcortical regions in WM performance. Furthermore, electrophysiological evidence suggests that systematic non-zero phase delays other than 180° are present, especially in the gamma band, between cortical regions and that a non-zero phase delay reflects the direction of communication between those cortical regions^14,21–23^. Moreover, the existence of this non-zero phase delay between involved cortical regions might contribute to compose an “optimal” brain state to perform specific cognitive tasks^14,20^.

Furthermore, recent findings have demonstrated that synaptic delays exist when the gamma band coupling entails communication directions^21,23,24^, and thereby lagged phase synchronization, which suppresses zero-phase lag synchronization, occurs^25^. Hence, it may be necessary to consider phase lag in applying multi-site tACS^14,20^. However, there is currently no proposed method that allows for determination of optimal injection currents to deliver desired phase delays other than in-phase (0°) and anti-phase (180°) over multiple cortical areas.

In this study, we developed multi-site multi-phase tACS (msmp-tACS), a modified version of tACS, based on the injection current optimization with finite element method (FEM) and complex least squares (CLS). The method can compute optimal electrode montages that induce an arbitrary phase delay over two selected cortical regions. Then, the feasibility of the proposed method was experimentally tested through a case study of high-gamma (80 Hz) msmp-tACS with optimized stimulation parameters to healthy human subjects performing visuospatial WM (VWM) tasks to investigate whether the phase delay induced over the bilateral intraparietal sulci (IPS) affected the laterality of VWM performance.

## Methods

### Computer simulations

#### Finite element head model

**Figure 1:**
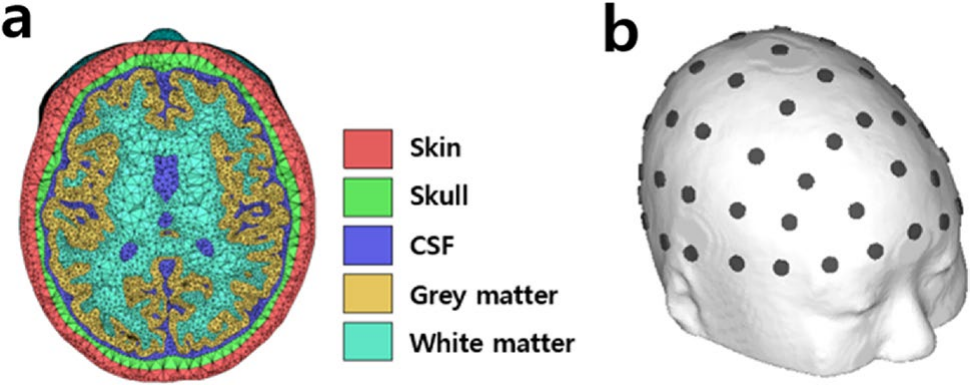
Finite element head model constructed using magnetic resonance imaging (MRI) data. (**a**) Cross-sectional view of the realistic human head model. (**b**) Locations of the 61 electrodes overlayed on the scalp surface.

A realistic human head model was constructed from the magnetic resonance imaging (MRI) data of a healthy male participant (24 years old, nationality: Korean). The T1-weighted MRI dataset was acquired using a 3 T MAGNETOM Trio Scanner (Siemens, Erlangen, Germany; spatial resolution = 0.8 mm × 0.8 mm × 0.8 mm). Subvolumes of the scalp, skull, gray matter, white matter, and cerebrospinal fluid (CSF) were segmented using an open software package, SimNIBS^26^. The segmented head model was then manually corrected using ITK-SNAP^27^. In addition, isolated meshes were eliminated to correct segmentation errors, and self-intersecting edges were corrected to improve the mesh quality using an in-house MATLAB 2019b (MathWorks, Natick, MA, USA) code. A detailed description of the segmentation error correction and mesh quality improvement can be found in a previous study^26^. The final head model consisted of approximately 1.6 million tetrahedral elements and 0.28 million nodes. A cross-sectional view of the human head model is presented in Fig. 1a. Sixty-one circular electrodes were attached to the head model using an in-house MATLAB 2018a code according to the international 10–10 EEG electrode system (see Fig. 1b for the electrode locations). The heights (2.5 cm) and radii (5 mm) of the electrodes were determined based on the electrodes used for the human experiments.

#### Problem formulation

For the constructed finite element head model, the electrostatic Laplace equation $\nabla \cdot \left( {\sigma \nabla V} \right) = 0$ (*V*: electric potential, *σ*: conductivity) was solved using FEM. To solve the FEM, an FEM solver embedded in COMETS2^28^ toolbox, the feasibility of which was validated by comparing its solution with an analytic solution^29^, was used. Dirichlet boundary conditions were initially applied as 1 V over the distal surface of the active electrode, with the distal surface of the return electrode being set as ground (*V* = 0). Then, using the two equations ${\mathbf{E}}\user2{ } = \user2{ } - \nabla {\text{V}}$ and ${\mathbf{J}}\user2{ }{ } = { }\sigma E$, where **J** and **E** denote current density and electric field, respectively, the boundary conditions were scaled for the current flowing in from the active electrode to be 1 mA. For the numerical computation, all the tissues were assumed to be isotropic, and the conductivity values of each tissue were set to 0.25 S/m (scalp), 0.015 S/m (skull), 1.79 S/m (cerebrospinal fluid; csf), 0.276 S/m (grey matter; gm), 0.126 S/m (white matter; wm), and 5.21 S/m (sponge electrode), as referenced in a previous study^30^. The active electrode was assumed to be located at Fpz. The return electrode was selected as one of the other electrodes, and the electric field was computed for every possible return electrode in turn. For each electrode pair, the electric field components normal to the cortical surface were computed. These pre-calculated electric field distributions were then used to compute cortical electric field distribution for an arbitrary injection current condition based on superposition theory.

#### Optimization

Using the computed electric fields, CLS was employed to determine the optimal injection currents from the return electrodes. To perform CLS, first, the regions of interest (ROIs) were manually segmented. For the preliminary simulation study, three ROI combinations were assumed as follows: 1) lateral intraparietal area (LIP) and frontal eye field (FEF); 2) left auditory cortex (LAC) and right auditory cortex (RAC); and 3) LIP, FEF, and dorsolateral prefrontal cortex (DLPFC). Then, the objective function that CLS minimized was expressed with complex values to convey phase information. For example, to simulate a stimulation with a phase difference of *ϕ* between two ROIs, the reference ROI would be assigned a uniform value of *A*cos(**0°**)** +** $jA$sin(**0°**), whereas the other ROI would be assigned *A*cos(*ϕ*)** +** $jA$sin(*ϕ*), and zero (0) would be assigned elsewhere. Here, *A* denotes the desired electric field amplitude (> 0) and *ϕ* denotes the desired phase difference in degrees. A positive value of *ϕ* would indicate the reference ROI leading the other, and a negative *ϕ* would indicate the opposite, where *ϕ* ranged from -180° to 180°. In our study, the desired amplitude (*A*) was assumed to be 0.3. Thus, the desired electric field distribution *f*(*A*,ϕ) over multiple ROIs was calculated as follows:

1 $$f(A,\phi ) = \left\{ {\begin{array}{*{20}l} {0.3\angle 0^\circ } \hfill & {if \Omega \in \Omega_{{ROI_{1} }} } \hfill \\ {0.3\angle \phi_{1} } \hfill & { if \Omega \in \Omega_{{ROI_{2} }} } \hfill \\ \vdots \hfill & \vdots \hfill \\ {0.3\angle \phi_{n} } \hfill & {if \Omega \in \Omega_{{ROI_{n} }} } \hfill \\ \end{array} } \right.$$

Note that the desired electric field property is expressed as phasor representation throughout the manuscript for clearer comprehension, i.e.,

2 $$A\cos (\omega t + \phi ) + jA\sin (\omega t + \phi ) = A\angle \phi$$

**Figure 2:**
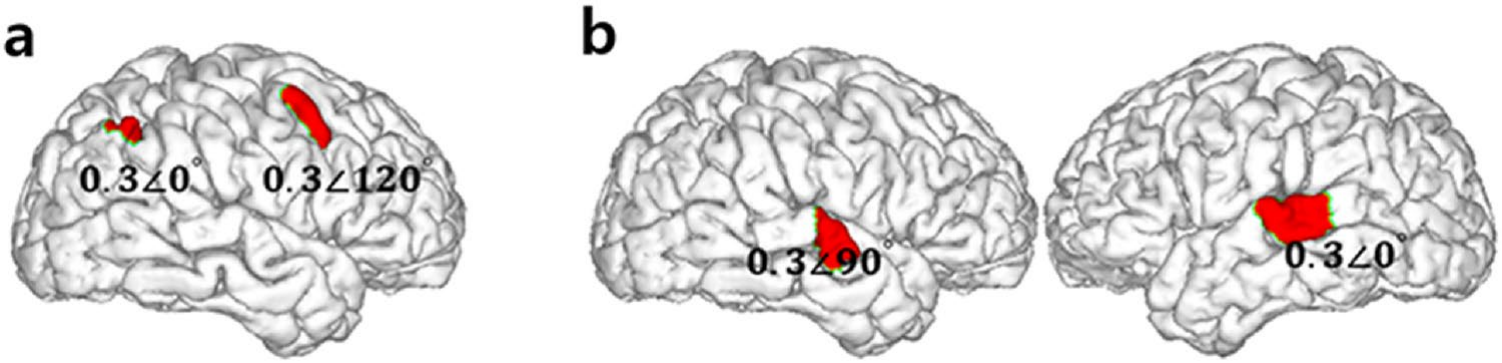
Two test region of interest (ROI) pairs and their respective objective function values. (**a**) Optimal stimulation phase of the lateral intraparietal area (LIP) leading the frontal eye field (FEF) by 120°. (**b**) Optimal stimulation phase of the left auditory cortex (LAC) leading the right auditory cortex (RAC) by 90°.

The phase delays chosen and assigned to the ROIs were arbitrary. In addition, we considered both the target frequency (ω) and time from the stimulation onset (*t*) to be deterministic, hypothesizing a linear system; therefore, they could be neglected in the process of CLS. The ROIs and their respective desired electric field properties for the first two ROI conditions (LIP-FEF and LAC-RAC) are shown in Fig. 2.

The injection current properties were then determined by solving for **x** in.

3 $$\mathbf{A}\mathbf{x}=\mathbf{b},$$

where **A** is a column-wise stacked complex matrix with its each column representing electric field over discretized space of cortex, **x** is an injection current vector, and **b** is a vector that represents the desired phase distribution (*f*(*A*,ϕ) over ROIs and zero elsewhere). As noted above, the solution that minimizes the squared error between the desired field and systematically realizable field, **x**_ls_, was obtained using a widely used least squares solution, where.

4 $${\mathbf{x}}_{\mathrm{ls}}=\mathrm{arg}\underset{\mathbf{x}}{\mathrm{min}}{\Vert \mathbf{b}-\mathbf{A}\mathbf{x}\Vert }_{2}={{(\mathbf{A}}^{\mathrm{H}}\mathbf{A})}^{-1}{\mathbf{A}}^{\mathrm{H}}\mathbf{b}.$$

After obtaining the CLS solution, which determines the injection current properties of the return electrodes, the injection current of the active electrode was calculated as the sum of the injection currents of the return electrodes multiplied by -1. The feasibility of this computation involving the linearity of electric fields is well described in a literature^31^. Finally, the maximum current amplitude of a single return electrode and the amplitude of the active electrode were limited to 2 mA and 4 mA, respectively, considering safety considerations.

**Figure 3:**
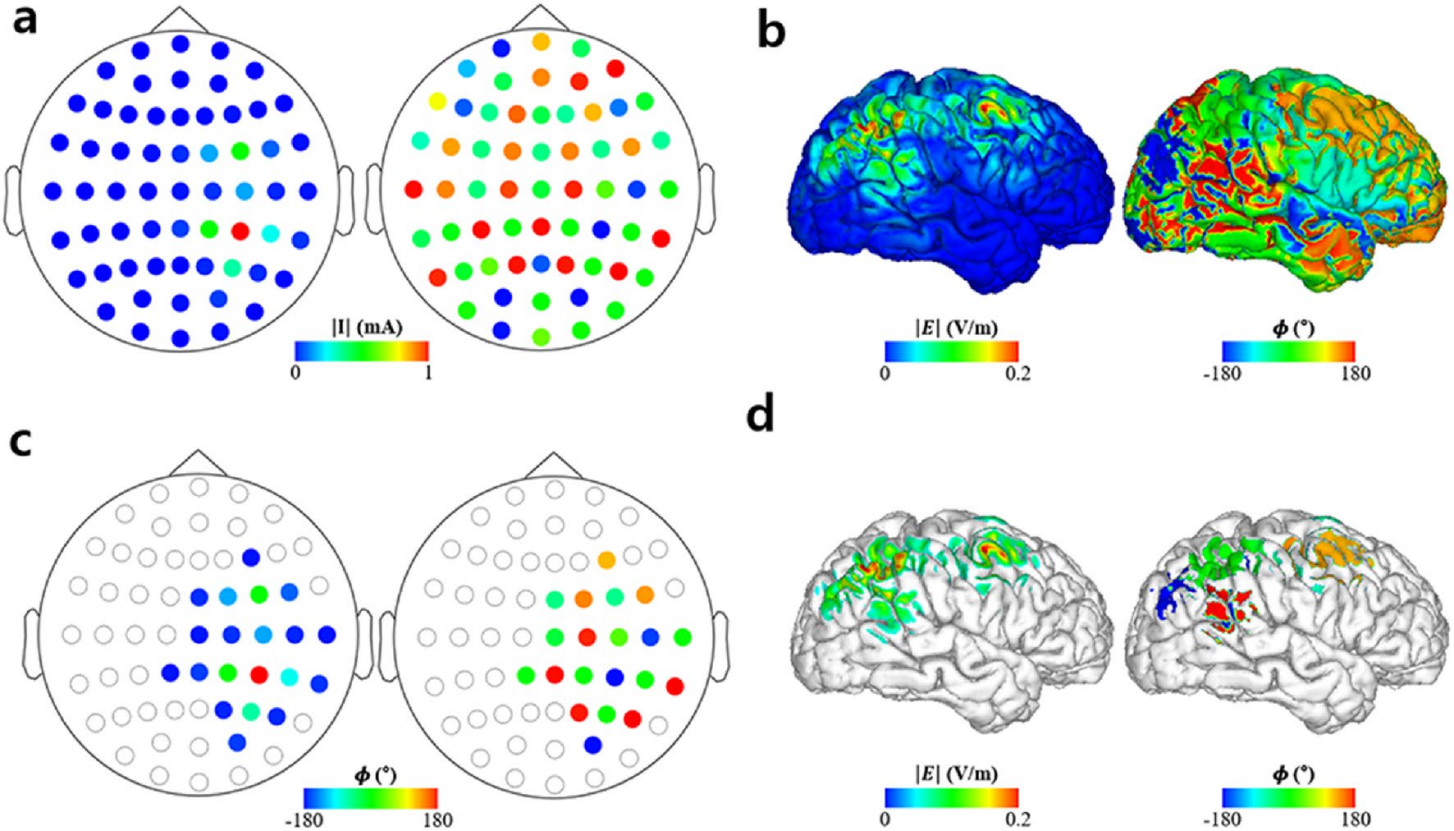
Optimization results of 61-channel montage when the region of interests (ROIs) were the lateral intraparietal area (LIP) and frontal eye field (FEF) with an optimal stimulation phase of the LIP leading the FEF by 120°. (**a**) Optimal injection current amplitude (left) and phase (right) distributions for all 61 channels. (**b**) Electric field amplitude (left) and phase (right) distributions under the optimal injection current condition. (**c**) Optimal injection current amplitude (left) and phase (right) condition of electrodes exceeding 0.1 mA in the injection current amplitude. (**d**) Electric field amplitude (left) and phase (right) distributions under the optimal electrode condition over cortical regions where the electric field amplitude was greater than 0.05 V/m.

**Figure 4:**
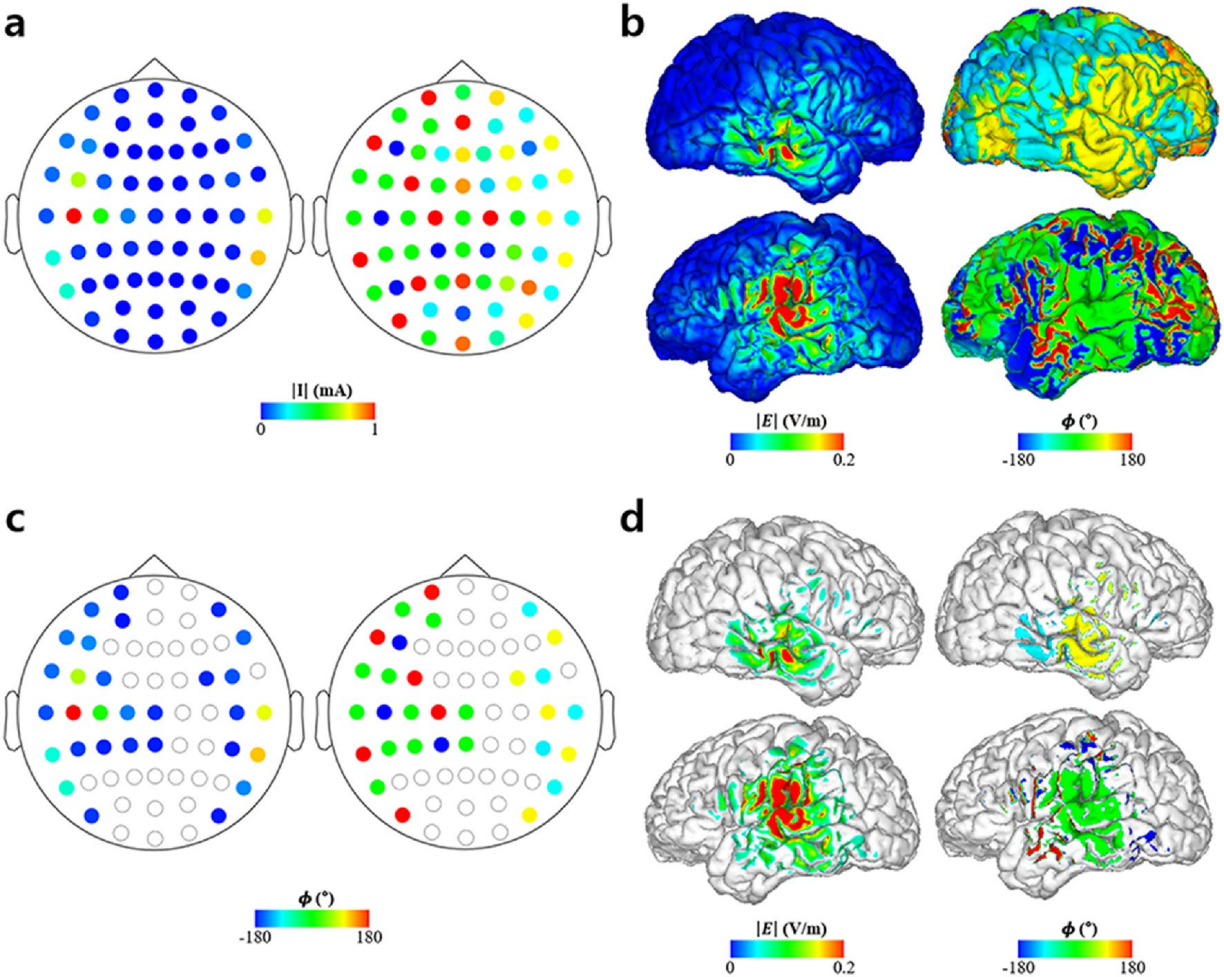
Optimization results when the region of interests (ROIs) were the left auditory cortex (LAC) and right auditory cortex (RAC) with an optimal stimulation phase of the LAC leading the RAC by 90°. (**a**) Optimal injection current amplitude (left) and phase (right) distributions for all 61 channels. (**b**) Electric field amplitude (left) and phase (right) distributions under the optimal injection current condition. (**c**) Optimal injection current amplitude (left) and phase (right) condition of electrodes exceeding 0.1 mA in the injection current amplitude. (**d**) Electric field amplitude (left) and phase (right) distributions under the optimal electrode condition over cortical regions where the electric field amplitude was greater than 0.05 V/m.

**Figure 5:**
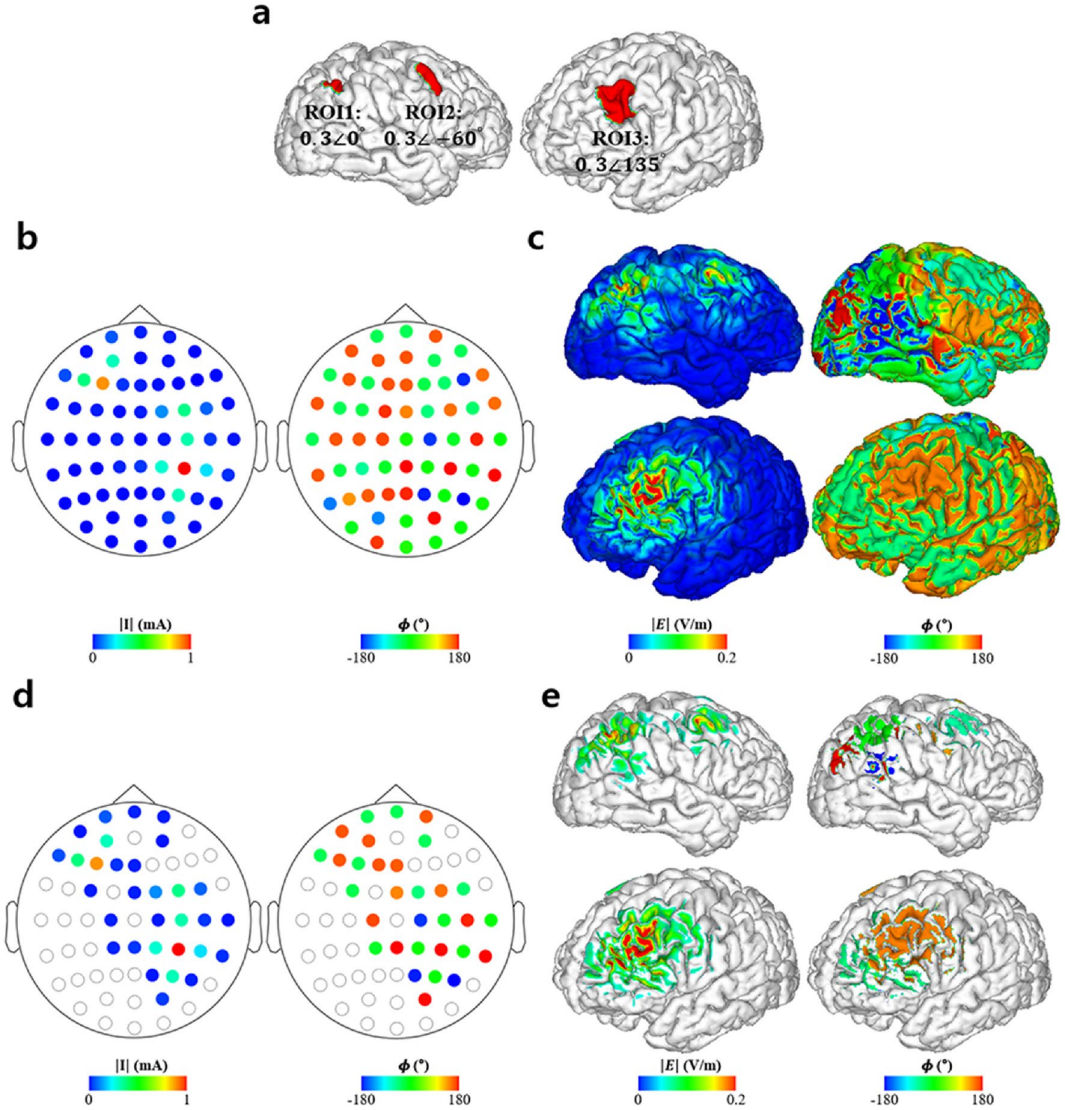
Optimization results for the simultaneous stimulation of the three cortical regions. (**a**) Region of interests (ROIs) and their respective values representing desired electric field property (**b**) Optimal electrode amplitude montage for all 61 channels (left) with a 0.1 mA cutoff (right). (**c**) Optimal electrode phase montage for all 61 channels (left) with a 0.1 mA cutoff (right). (**d**) Electric field amplitude distribution under the optimal electrode condition (left) and distribution of the electric field over 0.05 V/m (right). (**e**) Electric field phase distribution under the optimal electrode condition (left) and distribution of the electric field over 0.05 V/m (right).

The simulations for validation of the proposed methods are depicted in Figs. 3, 4, 5. Figure 3 shows the optimal injection current conditions and cortical electric field property distribution with the optimal stimulation phase difference of the LIP leading the FEF by 120°. Figure 3a depicts the optimal injection current conditions with amplitude and phase properties illustrated in the left and right columns. Likewise, Fig. 3b depicts the electric field properties over the cortex with the amplitude and phase distributions represented by the left and right columns, respectively. Figure 3c represents the electrode conditions of electrodes with injection current amplitudes greater than 0.1 mA. Similarly, the electric field properties of cortical regions with 0.05 V/m for an electric field amplitude are shown in Fig. 3d. The left and right columns of Fig. 3c,d, respectively, show the amplitude and phase distributions on the cortex, respectively. The cutoffs for the injection current amplitude and electric field amplitude over the cortex (i.e., 0.1 mA and 0.05 V/m) were chosen arbitrarily just for visualization purposes. The peak electric field induced over the LIP and FEF was 0.24 and 0.27 V/m, respectively.

Furthermore, to analyze the stimulation phase, we computed the stimulation phase, which was defined as the median phase over the half-maximum area of the ROIs. The median phase over the half-maximum area was 2.92° at the LIP and 117.20° at the FEF, which can be interpreted to indicate that the stimulation phase of the LIP led the FEF by 114.28°. The difference between the desired phase delay (120°) and the stimulation phase delay (114.28°) was just 5.72°.

The optimal electrode condition and electric field properties over the cortex with the optimal stimulation phase difference of the LAC leading the RAC by 90° is shown in Fig. 4. The maximum electric field over the LAC and RAC were 0.41 and 0.21 V/m, respectively. Finally, the median phases over the half-maximum area in each ROI were 0.03° at the LAC and 90.21° at the RAC, which yielded a difference of 90.18°, as illustrated in Fig. 4d. It was just a 0.18° lag to the optimal stimulation phase difference of 90°.

Finally, we further tested the feasibility of our method assuming three ROIs: LIP (0°), FEF (-60°), and DLPFC (135°). Angles in the parentheses denote the optimal stimulation phases for each region, as described in Fig. 5. Using the optimal electrode montage, the three regions were successfully stimulated simultaneously (Fig. 5d,e), with maximum electric fields of 0.24 V/m (LIP), 0.22 V/m (FEF), and 0.37 V/m (DLPFC) over each of the ROIs. Furthermore, the median phases over the half-maximum area of each ROI were -3.11°, -57.34°, and 135.00° over the LIP, FEF, and DLPFC, respectively. Consequently, the median phase differences reference to the LIP were -54.23° for the FEF and 138.11° for the DLPFC, with 5.77° and 3.11° lag to the optimal stimulation phases of FEF and DLPFC, respectively.

### Msmp-tACS

#### Participants

Eighteen healthy, right visual hemifield dominant volunteers (7 men and 11 women, age: 23.41 ± 2.90) participated in the msmp-tACS experiments. While there is no evidence that ocular dominance affects the VWM capacity of visual hemifields, we selected participants who had right ocular dominance for homogeneity. Individuals with any identifiable neurological disorder, head injury, or any personal or family history of psychiatric illness were excluded. The study protocol was approved by the Institutional Review Board of Hanyang University, South Korea (IRB No. HYU-2020–010). All methods were performed in accordance with the Declaration of Helsinki. Two participants were excluded from the analyses because they could not complete the protocol.

#### Behavioral task

**Figure 6:**
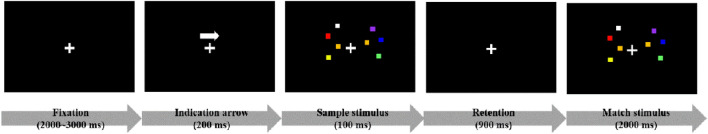
Match to delayed sample task for visuospatial working memory. A fixation period of 2000–3000 ms is followed briefly by the indication arrow (200 ms), which informs participants of the hemifield that they should memorize. After the indication arrow disappears, a sample stimulus, which is an array of squares, is presented for 100 ms on both hemifields, which are separated by a fixation cross. A retention period lasting for 900 ms follows, and a match stimulus is subsequently presented for 2000 ms. During the presentation of the match stimulus, participants are asked to indicate whether the colors of the squares on the hemifield indicated by the indication arrow matched the colors of sample stimulus.

A visual delayed match-to-sample task was employed^32^, in which each trial consisted of a fixation period (2000–3000 ms), indication arrow (200 ms), sample stimulus (100 ms), retention period (900 ms), and response stimulus (2000 ms); the duration of each stage is shown in parentheses (see Fig. 6). Throughout the task, a fixation cross was displayed at the center of the screen. After the initial fixation, an arrow pointing either left or right appeared for 200 ms above the fixation cross. Once the indication arrow disappeared, two sets of square arrays were displayed on the left and right sides of the fixation cross for 100 ms. All the squares in each array had different colors, and the number of squares in the two arrays displayed on both sides of the fixation cross were the same (4, 5, or 6). The squares were 0.65° × 0.65° in size, and the minimum distance from the center of a square to another was 2°. Each array of squares was presented within a 9.8° × 7.3° region and did not overlap with the fixation cross. To achieve this, participants were asked to fix their head on a chinrest located 60 cm away from the screen. Furthermore, the During this period, the participants were instructed to memorize the colors of the square array on the side that had been indicated by the arrow. After the offset of the sample stimulus presentation, a short retention period (900 ms) followed. Another square array, the response stimulus, was subsequently displayed on both sides of the fixation cross for 2000 ms. During this period, the participants were instructed to identify whether the colors on the array of squares located in the side that had been indicated by the arrow were the same as those presented during the sample stimulus period by pressing a button on the response pad.

The total number of trials in one experimental session was 120, and these were divided into six blocks of 20 trials. The six blocks were categorized into three sets of two blocks, with each set containing four, five, or six squares in a square array. The number of squares in an array corresponded to the task load. To minimize a potential learning effect, the six blocks were randomly selected from 18 different blocks (six blocks for each task load) using e-Prime 3.0 (E-Prime Psychology Software Tools Inc., Pittsburgh, USA). Of the 20 trials in each block, ten trials were left hemifield trials (indicated by the arrow pointing left), whereas the other ten trials were right hemifield trials (indicated by the arrow pointing right); 10 trials for each visual hemifields consisted of five match and five mismatch trials.

To quantify the VWM capacity, the K-value, defined as ‘load × (accuracy – miss rate)’, was computed. The K-value considers the task load and better represents WM capacity than accuracy^32^. In addition, the weighted average d’ values were computed. The d’ value is defined as ‘Z_hit_ – Z_FA_’, with FA indicating the false alarm rate^5,33^. The d’ values were computed for each load, multiplied by the corresponding load, and were then averaged.

Additionally, the lateralization index (LI) was calculated to analyze the laterality of the WM performance of the visual hemifields using the following equation:

5 $$\mathrm{LI}=\frac{{\mathrm{M}}_{R}-{\mathrm{M}}_{\mathrm{L}}}{{\mathrm{M}}_{\mathrm{R}}+{\mathrm{M}}_{\mathrm{L}}},$$

where M_R_ and M_L_ denote the measurements (K-values or load weighted average of d’ values) computed from right and left hemifield trials, respectively.

Finally, to analyze how the participants with different baseline performances responded to the stimulation, the aforementioned analyses for K-values, weighted d’, and LI computed using K-values and the weighted d’ were conducted for two groups separated according to the baseline performance: a low LI group and a high LI group under L0R0 stimulation, which was the baseline condition in our study. The threshold for determining the low and high LI groups was the median LI under the L0R0 condition, with participants who performed to the exact median LI being classified into the high LI group.

#### Msmp-tACS Protocol

Participants underwent three stimulation sessions, in which the desired phase delays induced by msmp-tACS over the bilateral IPS were in-phase (denoted by ‘synchronized’ condition), right IPS lagged the left IPS by 90° (denoted by ‘LR’ condition), and vice versa (denoted by ‘RL’ condition). A 90° delay of 80 Hz is equivalent to 3.125 ms. Current stimulation was delivered by Starstim 8 (Neuroelectrics Inc., Barcelona, Spain). Each stimulation session was at least 72 h apart. Before participating in the first of the three stimulation sessions, participants went through a hole-in-the-card test^34^ at a distance of 60 cm (equivalent to the distance to the monitor displaying the behavioral tasks) to determine their dominant eye. Current stimulation was delivered through eight electrodes attached over the parietal and occipital areas according to the international 10–10 EEG system (P3, Pz, P4, PO3, PO4, PO7, Oz, and PO8). The injection current conditions for each stimulation condition were determined using FEM-based field simulation and the CLS algorithm, as described earlier. The ramp up and down periods were 30 s, and the stimulation was aborted 1 min (including the ramp down period) after the task was completed. Additionally, the safety system of Starstim-8 was applied in the protocol, which aborts stimulation if impedance of 20 kOhm is reached at any electrode; however, there were no abortion due to the safety protocol. The behavioral task was performed while the stimulation was being delivered, starting 5 min after stimulation onset. One session of the behavioral task (six blocks) was completed for each stimulation condition. The order of the stimulations was randomized and double blinded. Of the 16 participants who completed all three stimulation sessions, none reported adverse effects, such as burning, itching, and hurting sensations or visual phosphenes.

#### Statistical analysis

To assess the effect of the stimulation on the K-values and d’ values of the left and right visual hemifield trials, a two-way, repeated measures ANOVA (rmANOVA) was performed for the within factors ‘hemifield’ and ‘stimulation condition’. One-way rmANOVA and post-hoc analysis of a paired-sample t-test were also performed in order if required. Furthermore, the K-values and d’ values for each visual hemifield were separately evaluated using a one-way rmANOVA. Finally, if post-hoc analysis was performed, the final p-values were corrected using the method of false detection rate (FDR). For the LI, a one-way rmANOVA was performed for the within factor ‘stimulation condition’. Post-hoc analysis with a paired-sample t-test was performed in order if necessary. Finally, to test the difference in performance between the high LI and low LI groups, Friedman’s test, Wilcoxon’s rank sum and signed rank test, if necessary, was conducted for K-values and weighted d’ values of different hemifields under each stimulation condition, since the group size was small (n = 8). All the reported final p-values were corrected using FDR. SPSS 22 was used for all the statistical analyses except for the FDR correction, which was done using MATLAB 2019b.

### Ethical approval

All the participants were provided with a detailed explanation of the experimental protocols and signed a written informed consent form. The study protocol was approved by the Institutional Review Board of Hanyang University, South Korea (IRB No. HYU-2020–010).

## Results

### Computer simulation

**Figure 7:**
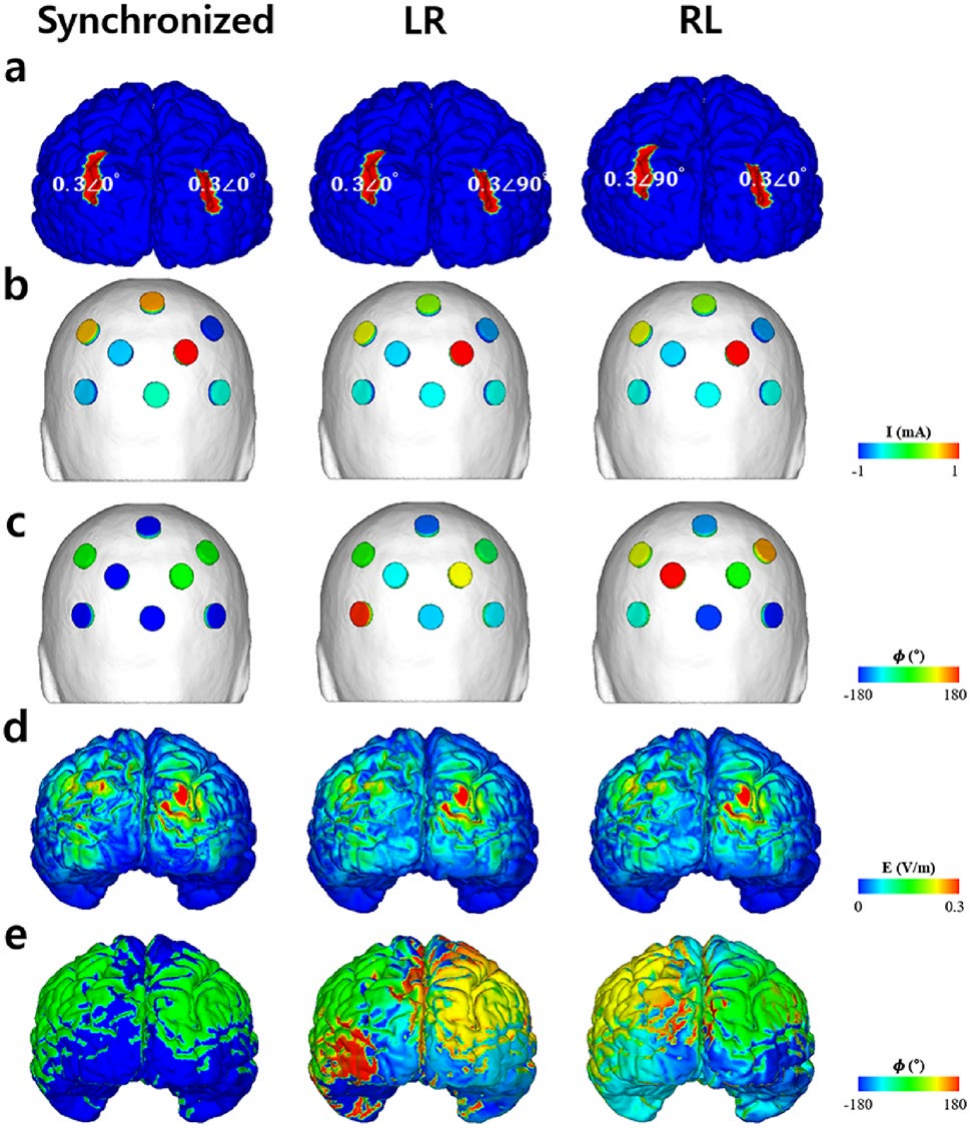
Region of interests (ROIs) and optimal injection current properties for each stimulation condition. (**a**) Segmented ROI (bilateral intraparietal sulcus) and desired stimulation condition, (**b**) optimal injection current amplitudes for each stimulation condition, (**c**) optimal injection current phase properties for each stimulation condition, (**d**) electric field amplitude distribution over cortex under the optimal condition, (**e**) electric field phase distribution over the cortex under the optimal condition.

The optimal injection current properties and electric field induced over the cortex under the optimal conditions were computed using FEM and CLS. Figure 7 illustrates the optimal injection current properties for the three stimulation conditions (synchronized, LR, and RL) and electric field distributions on the cortex under the optimal conditions. For the optimal montages of LR and RL, the amplitudes of the injection currents were identical (Fig. 7b), whereas the phases of the injection currents were different (Fig. 7c). This led to the same electric field amplitude distribution over the cortex (Fig. 7d), albeit different phase distributions (Fig. 7e). In contrast, for the optimal montage of the synchronized condition, the optimal injection currents were different from the other two cases (LR and RL). As shown in Table 1, the maximum electric field amplitude was 0.35 V/m under the synchronized condition, whereas it was 0.31 V/m under the other conditions. For the phase distributions, the difference between the median phase over the half-maximum areas (areas with electric field amplitudes higher than half of the maximum electric field amplitude over each ROI) of the left and right IPS was 0°, -90.97, and 87.55° for the synchronized, LR, and RL montages, respectively. The error between the median phase differences and the desired phase delays was less than 2.7%, as shown in Table 1.

**Table 1:** Properties of the electric field delivered over the cortex by each stimulation condition.

conditions	synchronized	LR	RL
E_max_ (V/m)	0.45	0.4	0.4
${E}_{max}^{rIPS}$(V/m)	0.34	0.31	0.31
${E}_{max}^{lIPS}$(V/m)	0.30	0.29	0.29
${\boldsymbol{\varphi }}_{max}^{rIPS}$(°)	0	89.66	− 1.21
${\boldsymbol{\varphi }}_{max}^{lIPS}$(°)	0	− 1.31	86.34
Phase difference (°)	0	90.97	87.55
Error (%)	0	1.1	2.7

### Msmp-tACS

The main indices used for the analysis were the K-value, weighted average of d-prime values (d’), and LI. The K-value is an index that accounts for both the load and accuracy of a WM task and represents sensitivity, while the d’ value represents the sensitivity and normalized overall performance of the task. Both values were computed for each visual hemifield. The LI is a normalized difference between an index (either the K-value or d’) of visual hemifields, indicating to what extent the WM performance of the right hemifield is more dominant than that of the left hemifield (i.e., positive values implicate better performance for the right hemifield).

**Figure 8:**
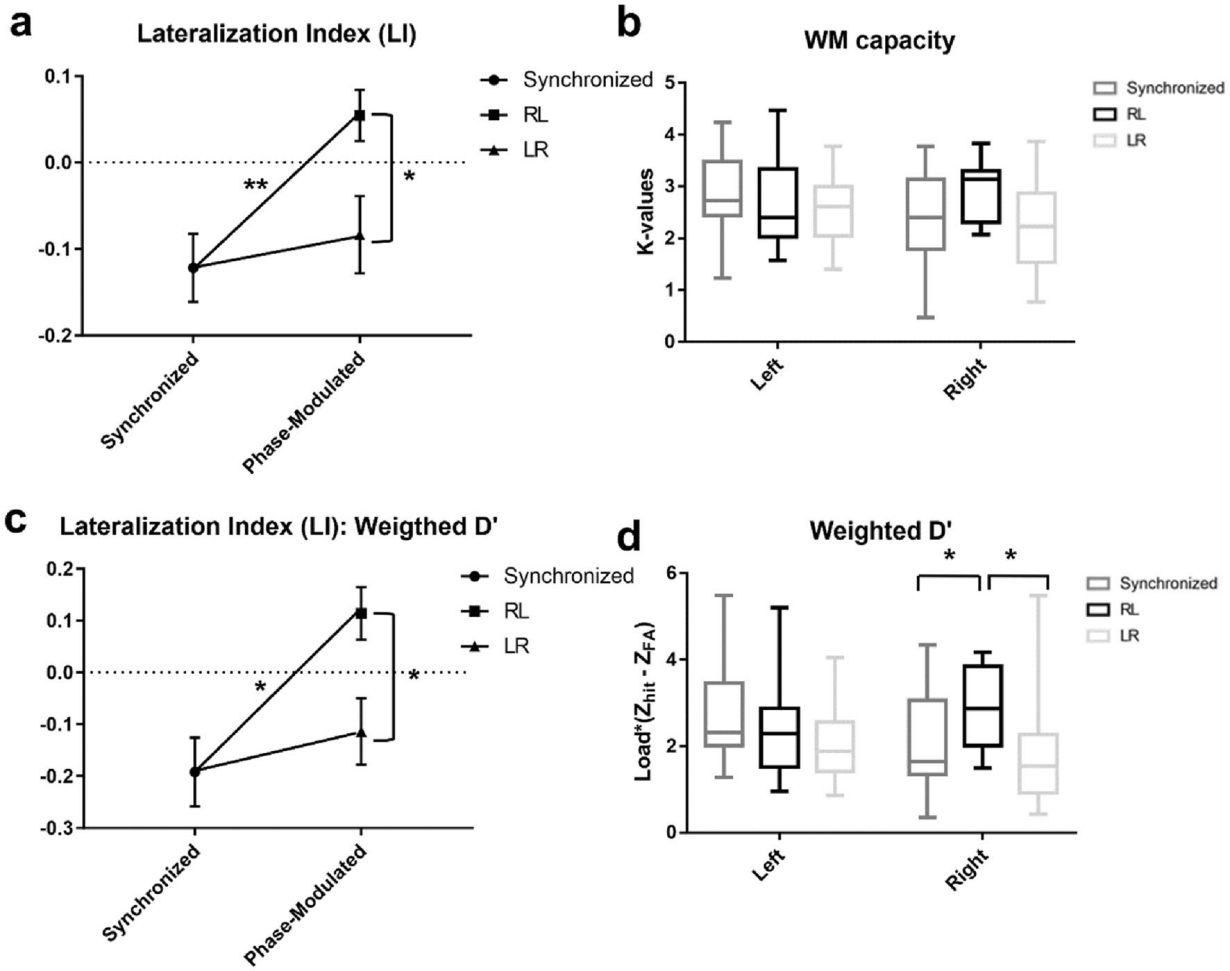
Behavioral performance under each stimulation condition. (**a**) lateralization index (LI) computed using K-values, (**b**) visual hemifield specific K-values under each stimulation condition, (**c**) lateralization index (LI) computed using the weighted average of d’ values, (**d**) visual hemifield specific the weighted average of d’ values under each stimulation condition (* p < 0.05; ** p < 0.01).

The dominance of VWM capacity shifted toward the right visual hemifield, as evidenced by one-way rmANOVA and a subsequent paired-sample t-test; a significant effect of a non-zero phase delay on the LI was found (F_2,__13_ = 9.86; *p* < 0.01, Fig. 8a). Specifically, a post-hoc paired-sample t-test further revealed that the effect was significant only under the RL condition, (t_15_ = 3.95, *p* = 0.004) but not under the LR condition (t_15_ = 1.09, *p* = 0.29) compared with the synchronized condition. Considering the two non-zero phase delay stimulations, the RL condition showed a higher effect than the LR condition (t_15_ = 3.07, *p* = 0.012). All p-values are corrected using the FDR. These results suggest a shift in the VWM performance toward the right visual hemifield under the RL condition. Notably, a statistically significant left- hemifield dominant lateralization of VWM capacity was present under the synchronized stimulation condition, as evidenced by one-sample t-tests of the LI against zero (t_15_ = -3.08, *p* < 0.01, mean ± standard error: -0.122 ± 0.04). However, the stimulation effect on the LI was only marginal under phase-modulated conditions (LR: t_15_ = -1.87, *p* = 0.08, -0.084 ± 0.04; RL: t_15_ = 1.84, *p* = 0.08, 0.054 ± 0.03).

Furthermore, a two-way rmANOVA performed for the K-values with the within factors ‘hemifield’ and ‘stimulation condition’ showed significant effect of the stimulation condition × hemifield interaction (F_2,__10_ = 7.3, *p* = 0.02), but not for the within factor ‘stimulation condition’ (F_2,__10_ = 2.36; *p* = 0.15) and ‘hemifield’ (F_2,__10_ = 2.53; *p* = 0.13). Subsequent one-way rmANOVA conducted for the K-values specific to the visual hemifield with the within factor ‘stimulation condition’ showed significant effect only on the right hemifield trials (left hemifield K-value: F_2,__13_ = 1.3, *p* = 0.27; right hemifield K-value: F_2,__13_ = 5.66, *p* = 0.03). The post hoc analysis indicated significant increase in right hemifield K-value under RL condition only when compared to the LR condition (t_15_ = -3.22, *p* < 0.01). This implies that the increase in the LI under the RL condition was more likely due to an increase in the right hemifield WM capacity rather than due to a decrease in the left hemifield WM capacity, although a differential effect of lateralization occurred across participants. The K-values of the left and right visual hemifields under each stimulation condition are depicted in Fig. 8b.

In addition to the K-values, we calculated the weighted average of the d’ values for each stimulation condition to evaluate the reliability of task performance. The d’ values are computed by subtracting the Z scores of the false alarm rate from the Z scores of the hit rate. The computed d’ values were weighted by the load of each trial and then averaged. The LI was also computed using the weighted average of the d’ values.

The results using LI values computed using weighted d’ showed the same trend as the LI values computed using K-values (Fig. 8c,d). One-way rmANOVA indicated a significant effect of the within factor ‘stimulation condition’ (F_2,__13_ = 7.18, *p* = 0.02). A consequent paired-sample t-test also showed the same trends with the LI computed using the K-values (synchronized-RL: t_15_ = -3.28, p = 0.02; LR-RL: t_15_ = -3.05, *p* = 0.03; synchronized-LR: t_15_ = 0.71, *p* = 0.28). Moreover, the one-sample t-tests against zero indicated that the normalized VWM performance was significantly lateralized toward the left and right the under synchronized and RL conditions, respectively; however, no significance was found under the LR condition (synchronized: t_15_ = -2.9, *p* = 0.01; LR: t_15_ = -1.78, *p* = 0.1; RL: t_15_ = 2.23, *p* = 0.04). The mean and SE of the LI using the weighted average of d’ were -0.19 ± 0.07, -0.11 ± 0.06, and 0.11 ± 0.05 for the synchronized, LR, and RL conditions, respectively.

To further analyze the weighted d’, a two-way rmANOVA was performed with the within factors ‘stimulation condition’ and ‘hemifield’ for the measure weighted d’. The test revealed the same trend with the analyses of K-values, showing significant for the hemifield × stimulation condition interaction, and within factor ‘stimulation condition’ (F_2,__10_ = 5.07, *p* = 0.04 for hemifield × stimulation condition interaction; F_2,__10_ = 5.68, *p* = 0.03 for the within factor ‘stimulation condition’; and F_2,__10_ = 0.98, p = 0.34 for the within factor ‘hemifield’). Subsequent repeated measures one-way rmANOVA and paired t-test confirmed that the weighted average of d’ increased significantly for the right visual hemifield trials during the RL stimulation condition compared with the other two stimulation conditions (rmANOVA: F_2,__13_ = 7.42, *p* = 0.02; t-test: t_15_ > 3.05, *p* < 0.05). However, no significant effect of the within factor ‘stimulation condition’ was found for the left visual hemifield trials (F_2,__13_ = 2.9, *p* = 0.11).

Additionally, the overall accuracy, overall reaction time (RT), load-specific accuracy, and correct rejection rate (CRR) were analyzed with respect to visual hemifields. Repeated measures two-way rmANOVA performed for RT, load-specific accuracy and CRR with the within factors ‘hemifield’ and ‘stimulation condition’, while repeated measures one-way rmANOVA was performed for the overall accuracy. For RT, a simple linear transform used to normalize the data, by subtracting minimum and dividing by range of each RT dataset. For each measure, no significant interaction between the factor ‘stimulation condition’ and ‘hemifield’ (RT: F_2,__10_ = 0.34; *p* = 0.72; CRR: F_2,__10_ = 2.01, *p* = 0.15) was observed. Also, no significance was observed for the overall accuracy (F_2,__13_ = 2.38, *p* = 0.11). Likewise, an analysis performed on accuracy of each load did not show any significant interaction of stimulation condition × hemifield (load 4: F_2,__10_ = 2.87; *p* = 0.08; load 5: F_2,__10_ = 3.87; *p* = 0.06; load 6: F_2,__10_ = 1.5; *p* = 0.24).

**Figure 9:**
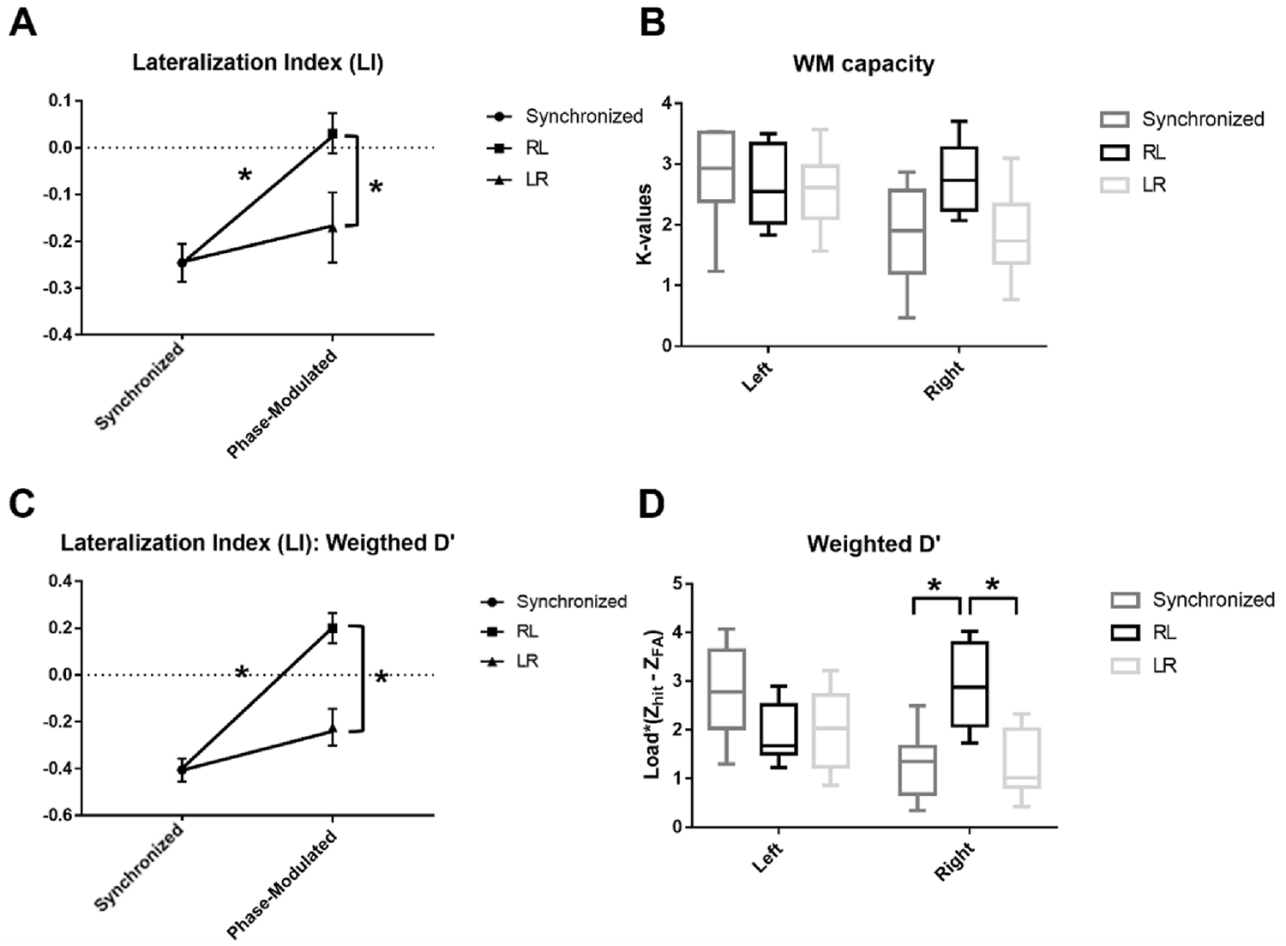
Behavioral performance of low LI group under each stimulation condition. (**A**) lateralization index (LI) computed using K-values,**B**) visual hemifield specific K-values under each stimulation condition, (**C**) lateralization index (LI) computed using the weighted average of d’ values, (**D**) visual hemifield specific the weighted average of d’ values under each stimulation condition (*p < 0.05).

Furthermore, Friedman’s test showed a significant difference only for the behavioral performance of low LI groups. Specifically, significant difference was observed for LI computed using K-value (χ^2^ = 9.75, *p* < 0.01), K-values of right visual hemifield trial (χ^2^ = 6.25, *p* = 0.044), LI computed using weighted d’ (χ^2^ = 13, *p* < 0.01), and weighted d’ values of right visual hemifield trials (χ^2^ = 7.75, *p* = 0.02) for the low LI group. Subsequent Wilcoxon’s signed rank test exhibited similar results to the total group of participants, as LI computed using K-value was significantly larger under RL stimulation compared to synchronized stimulation (*p* = 0.02) and LR stimulation (*p* = 0.035), but no significant difference was observed for the K-values of right visual hemifield trials. Additionally, the LI computed using K-value was significantly smaller than zero only under synchronized stimulation (*p* < 0.01). Finally, both LIs computed using weighted d’ and weighted d’ value itself were significantly larger under RL stimulation compared to synchronized stimulation (LI: *p* = 0.01, weighted d’: *p* = 0.035) and LR stimulation (LI: *p* = 0.01, weighted d’: *p* = 0.035). Interestingly, LI computed using weighted d’ was significantly smaller than zero under both synchronized (*p* < 0.01) and LR stimulation (*p* = 0.02), and significantly larger than zero under RL stimulation (*p* = 0.04). The behavioral performance of low LI group is depicted in Fig. 9.

**Figure 10:**
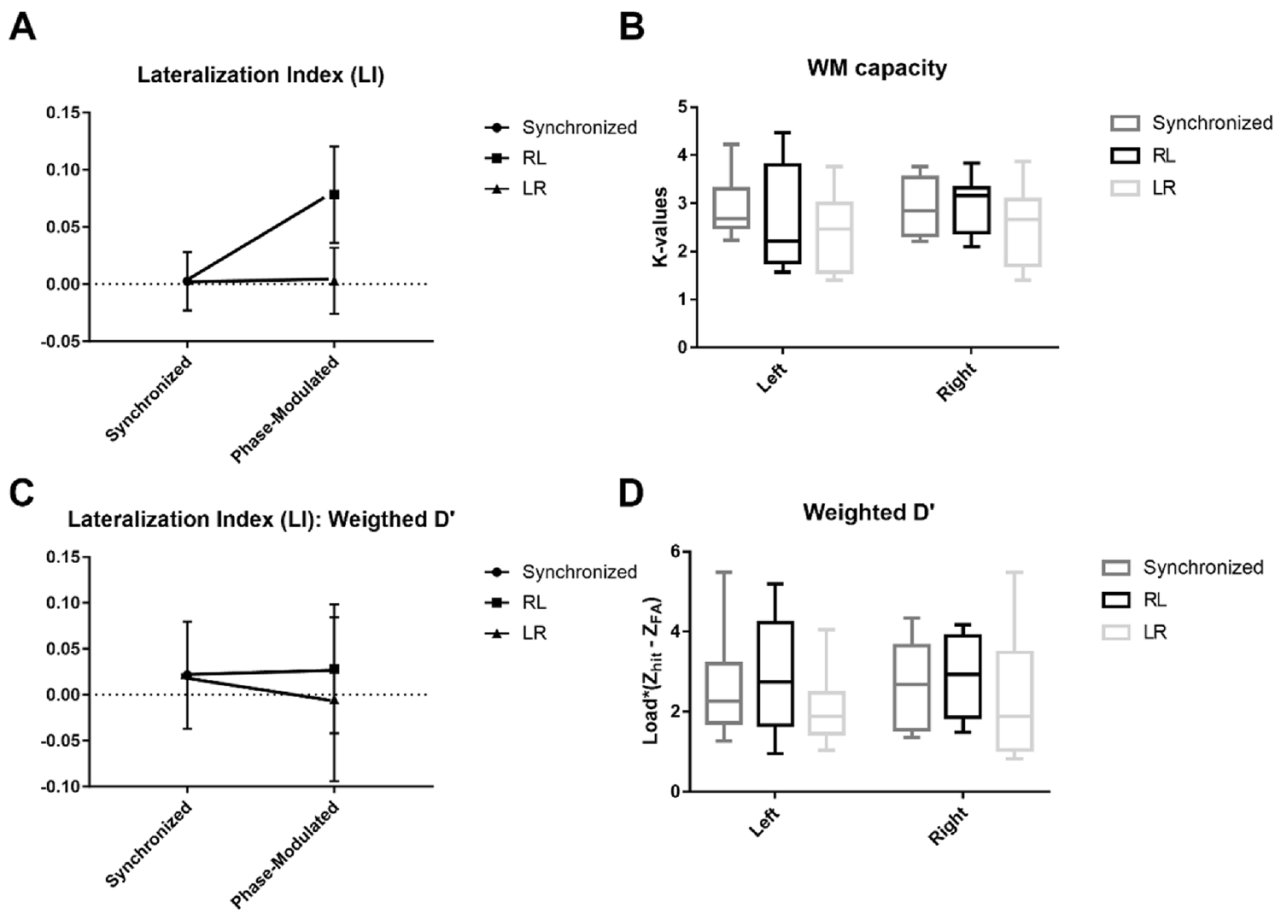
Behavioral performance of high LI group under each stimulation condition. (**A**) lateralization index (LI) computed using K-values, (**B**) visual hemifield specific K-values under each stimulation condition, (**C**) lateralization index (LI) computed using the weighted average of d’ values, (**D**) visual hemifield specific the weighted average of d’ values under each stimulation condition.

Meanwhile, the high LI group did not show any statistical significance neither between stimulation conditions (LI: χ^2^ = 1.75, *p* = 0.42; weighted d’: χ^2^ = 0.75, *p* = 0.68), nor against zero (lowest *p* value: 0.15, between 0 and LI under RL condition; one-sample t-test) at any stimulation conditions. The behavioral performance of the high LI group is illustrated in Fig. 10.

**Figure 11:**
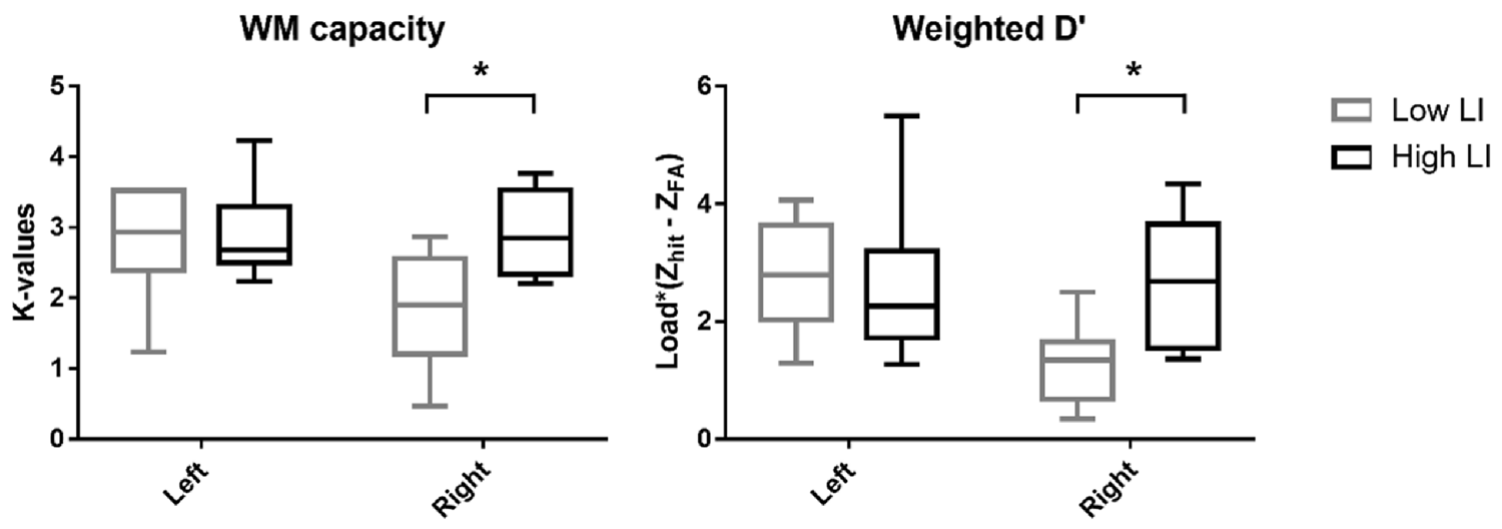
The baseline performance of the low and high LI groups. (*p < 0.05).

The main difference between high LI and low LI groups were performance of right visual hemifield trials under synchronized condition, as rank sum test revealed that low LI group exhibited significantly lower K-values and weighted d’ values (LI: *p* = 0.02, weighted d’: *p* = 0.01) under synchronized condition, which is the baseline performance, as depicted in Fig. 11. No other performances were significantly different between the two subgroups (lowest *p* value: 0.13, between K-values of right hemifield trials under LR condition). All the reported *p* values are FDR corrected if necessary.

## Discussions

In this study, we attempted to induce a systematic phase delay of the gamma band (80 Hz) in the bilateral parietal areas and to explore how such a phase delay modulates VWM performance. The stimulation frequency (80 Hz) was selected over 40 Hz, a conventional gamma tACS frequency, because 80 Hz affected VWM capacity significantly while 40 Hz did not^35^. To deliver focal msmp-tACS, we introduced a new method to simulate and optimize tACS protocols using complex values. This enabled the simultaneous representation of the phase and amplitude of the delivered electric fields. While “traveling wave tACS”^36^ has been developed and validated to enable tACS with phase lag, targeting specific cortical areas with desired phase delays requires additional experiments not reported in the study (i.e., location of return electrodes or optimal stimulation phases cannot be determined). Importantly, the traveling wave tACS reports electric fields distributed with spatial phase gradient where stimulation electrodes are not attached, and intensities of such cortical regions along the path of E-field is not usual targets for NIBS in human experiments. Nonetheless, traveling wave tACS is promising and more proven, and thorough validations made through the traveling wave tACS study is not present in our study. However, the facts that our method was an extended version of previous literatures and the validations of multi-phase tACS made on the traveling wave tACS study at least provides indirect evidence for feasibility of our method. To address the shortcoming, we performed a case study by implementing the proposed method to healthy participants.

As a result of the case study, we successfully induced the desired phase delays in the target cortical regions. Through 80 Hz msmp-tACS, we found that the lateralization of VWM performance was shifted toward the right visual hemifield when the stimulation phase of the right IPS (rIPS) led that of the left IPS (lIPS) by 90° (RL condition) compared with the synchronized or lIPS-leading-rIPS (LR) stimulation conditions. Additionally, by analyzing the behavioral performance of subgroups of participants based on their baseline performance, we found that the behavioral changes for different stimulation conditions were mainly due to relatively poor performance in right visual hemifield trials under synchronized condition. Indeed, stimulations mostly affected the low LI group, suggesting possible "ceiling effects". However, whether the observed LI is a true "ceiling" needs further investigation, as phase differences other than 90° might effectively increase or decrease LI.

Previous literature has emphasized synchronization (in-phase condition) or desynchronization (anti-phase condition) of cortical regions, stating that in-phase synchronization induced by tACS strengthens network entrainment^37^. Nevertheless, a zero-phase delay between cortical regions may not necessarily lead to an optimal network state or enhanced functional synchrony due to phase lags and neural transmission delays^38,39^. Likewise, a 180° phase delay between cortical regions may not connote that the involved cortical network is defunct or disturbed. Therefore, it is essential to explore changes in cognitive functions under such phase delays between functionally related cortical regions.

Upon applying msmp-tACS, the RL condition increased the LI of the VWM capacity compared with the other stimulation conditions. However, the LI did not differ between the LR and synchronized conditions. One possible explanation could be that at the “natural” state, or a state in which no stimulation is applied, the phase delay between the bilateral IPS would be somewhere between zero and the left IPS leading the right IPS by 90°. This speculation is in accordance with the findings of a previous dual-site tACS study^40^, which suggested that there might be an optimal phase delay between 0° and 180° of the gamma band at the bilateral parietal cortices is a naturally occurring feature during WM task performance. However, a sham group is necessary to observe the “natural” state, and thus we enrolled 18 new participants to perform the same task without stimulation. The sham group did not show the expected mean LI value in between the synchronized and LR condition (Figure S1, in Supplementary Material). Nonetheless, independent samples t-test results showed interesting results as the K-values of right hemifield trials were significantly higher compared to the sham group only under the RL stimulation condition, while a trend of higher left hemifield K-value was observed under synchronized condition compared to the sham group (Figure S2).

Our results indicate that the phase delay induced over the bilateral IPS caused a shift in lateralization of the VWM performance toward the right visual hemifield. Although contralateral bias of the parietal sites does exist when performing VWM tasks, both the bilateral parietal areas are considered responsible for the binding of VWM^41–43^. Especially, the bilateral parietal areas are reported to be activated when performing WM tasks with higher cognitive loads^44^. Overall, activation of the ipsilateral parietal sulcus is believed to be observed when additional resources are required^44^, making communication between bilateral parietal areas more vital when performing WM tasks with high cognitive loads. As abovementioned, such communication arises in the form of synchronized gamma waves; since systematic delays in the gamma band entail the direction of communication between the cortical regions^21–23^, we cautiously suggest that such a shift in the LI is a behavioral observation of the change in the directionality of communication between the bilateral parietal areas.

We selected 80 Hz, a frequency within the high gamma range, as the stimulation frequency for the msmp-tACS experiment based on previous studies^5,45,46^. It could be argued that a 90° phase difference at 80 Hz (approximately 3 ms) is too short of a time delay to claim any significance of intercortical phase delay on VWM performance. However, spurious synchronization within assemblies of neurons with a delay as small as 2 ms has been observed during the retention period of visual memory^47^. The study reported that as visual features are coded to a specific assembly of neurons, spurious synchronization with ± 2 ms phase lags were observed in concurrent assemblies. Furthermore, Besserve et al.^48^ reported stimulus-modulated phase delays of the high gamma band in the primate visual cortex during performance of visual tasks^49^. However, the role of such time-delayed, spurious synchronization of neurons on cognitive function is unclear at best. The reason for such controversy is that it is challenging to observe a designated phase delay during cognition due to individual variability and the nature of EEG. Indeed, while our stimulation condition presumably induced a 90° phase delay, it is possible that an "optimal" phase delay other than 90° exists between bilateral parietal areas during VWM performance. Thus, to develop and test a method to explore phase delay other than 0°and 180°, it was only feasible to target the phase delay that was not biased towards both 0° and 180° phase delay, which was 90°.

Furthermore, given the short delay of 3 ms between cortical regions, the behavioral results exhibited by msmp-tACS is unlikely to be the result of msmp-tACS modulating a direct transmission of signals during working memory task. While the previous literature, the traveling wave tACS, aims to target entire cortical pathway in between multiple cortical regions considered as a sender and a receiver, the proposed msmp-tACS targets more specific regions. Although it is possible to target entire pathway using msmp-tACS, our montage and design of the case study exclusively induces desired phase delay over bilateral IPS, but not the pathway that connects the two regions. Therefore, the putative underlying mechanism is more likely the altered functional connectivity between the bilateral IPS due to the intervention of msmp-tACS. Indeed, such short delay was reportedly related to lateralization of auditory function during the dichotic listening task^14^. In the future, msmp-tACS could be combined with imaging methods to investigate whether the systematic delays of gamma oscillation truly entail direction of communication between cortical regions.

In general, a weighted average of d’ values are not a common measure used to analyze VWM performance. Nonetheless, we computed this index since we wished to analyze the normalized performance of the task according to the difficulty of the task. Careful interpretation of the results indicates that the LI of both the weighted averages of the d’ and K-values were affected by the different stimulation conditions; however, the non-significance of the interaction condition × hemifield on the K-values implies that pd-tACS altered the dominance of the visual hemifield in VWM without significantly affecting the overall VWM capacity. This is reiterated by the statistical non-significance involving the accuracy of each load. Indeed, statistical significance was found for both the K-values and weighted average of the d’ values among the right visual hemifield trials but not among the left visual hemifield trials, but the significance was not consistent for the K-value. The weighted average of the d’ values stresses accuracy more than the K-values; therefore, the results again support the notion that phase delays over the parietal areas may not reflect higher cognitive loads. Nevertheless, they are critical for processing and storing visuospatial information and possibly prioritizing this information for processing.

The trend of the right visual hemifield trials being affected is feasible considering the flexible resource model. In the model, the contralateral bias of the bilateral parietal areas has been shown to be greater in the representation of items in the left visual hemifield^50,51^. Namely, since the stimulation was bilateral, the VWM performance of the right visual hemifield could be more prominently affected by our stimulation conditions. Indeed, further results on the hemifield-specific weighted average of d’ values support this idea: the weighted average of the d’ values of only the right visual hemifield significantly increased under the RL stimulation condition compared with the other two conditions, while no significant change was observed for the left visual hemifield trials.

Given the reports stating that asymmetries of activation levels and timing in the frontoparietal network are vital to VWM performance^52,53^, it could be argued that our results stem from the asymmetry of the electric field amplitude delivered to the bilateral IPS. Nonetheless, the maximum electric field amplitude was greater over the right IPS than over the left IPS under all three stimulation conditions. However, if the results were due to amplitude asymmetry, the LI would have been altered only by the synchronized condition in which the amplitude asymmetry was greatest. Hence, we believe that the shift in the LI is not related to the asymmetric electric field amplitude delivered over the bilateral IPS. In addition, the effect of the RL condition may originate from the difference in distributions of the electric field amplitude over the cortex under the RL and synchronized conditions. However, this is less likely due to the following reasons: (1) the LIs were not significantly different under the synchronized and LR conditions, and (2) the LI was higher under the RL condition than under the LR condition that displayed an identical electric field amplitude distribution over the cortex. Moreover, the RL/LR conditions delivered an electric field over the cortex that was sufficient to modulate oscillatory activity^54,55^. Therefore, it is reasonable to conclude that the shift in the lateralization of VWM capacity was the product of the phase lag between the bilateral IPS.

Given these results, the inter-regional phase delay between cortical areas could also play a significant role in the lateralization of other behaviors, such as right ear advantage or handedness. Granted, msmp-tACS could be a useful tool to explore the causality between phase asymmetries of cortical regions and behavioral lateralizations. Clinically, the right ear advantage is known to be significantly decreased in patients with schizophrenia with auditory-verbal hallucinations (AVHs)^56–58^ because of changes in the gamma band. However, both in-phase and anti-phase gamma tACS of the bilateral auditory cortices failed to modulate the right ear advantage^14^; utilizing msmp-tACS in the gamma range may enhance the right ear advantage, which in turn may potentially help to alleviate AVH symptoms.

In the stimulation experiment, the main reason the sham condition was absent was to eliminate the effect of the frequency of stimulation when comparing sham to stimulation. Namely, even if behavioral performances had been different under sham and other stimulation conditions, the observation would have been insufficient to conclude that the changes were strictly caused by phase delays induced by msmp-tACS. Instead of a sham condition, we implemented the synchronized stimulation condition, which actss as a “reference state” rather than the default or control state generally assumed by the sham condition.

A limitation of the current research is that our stimulation protocols were based on computer simulations; therefore, disparities could exist between the actual stimulation phase and the phase computed over the ROI with simulation. To minimize such simulation errors, individual MRI can be employed; however, the results could still be erroneous compared with the actual field induced over the cortex. Indeed, using a representative model for the case study may cause differential effects of stimulation for each participant. However, the phase distribution of the E-field is much less susceptible to individual variability in anatomy compared to the amplitude of E-field delivered over cortex. For example, electric field amplitude delivered over a region of interest will be affected by the distance of electrode from the cortex, geometrical feature of the ROI, thickness of skull, etc. However, phase is much less susceptible to these features, as amplitude of E-field does not necessarily correlate with induced phase lags. That is, if a relative phase of 0° and 90° is observed between site A and B, the phase delay should be consistent unless interfered by other current source, even if the E-field amplitudes are decreased over the sites. To further address this issue, we conducted an additional simulation study by applying the identical electrode condition used in our case study to a finite element head model constructed using an MNI Colin head model (MNI; Montreal, Canada). The results showed that the induced phase delays for LR and RL conditions were -97.82° and 86.91° (phase of rIPS subtracted from phase of lIPS), respectively, indicating that electrode condition determined by using representative model was valid in other head model. Additionally, the E-field amplitude was strong over rPIS and lIPS when simulated in the Colin head model (LR: 0.29 V/m for lIPS and 0.33 V/m for rIPS; RL: 0.29 V/m for lIPS and 0.38 V/m for rIPS). The result of the additional simulation is illustrated in Figure S3.

Finally, we omitted the anti-phase stimulation. It is possible that the anti-phase condition may drive lateralization in the other direction compared with the synchronized condition. Furthermore, current evidence is lacking regarding the actual or optimal phase delays necessary during synchrony of cortical regions. Therefore, the role of specific phase delays over brain regions, such as the condition where one ROI leads another by 45° and the anti-phase condition, on various cognitive functions could be investigated in future studies.

## Conclusion

In this study, we proposed a novel method, the msmp-tACS, which allows for stimulation of multiple brain regions with desired phase delays. In an effort to represent phase information in the simulation study, we employed complex values. Also, the optimization method was modified from LS to CLS. The computer simulations confirmed that focal electric fields with desired phase delays could be delivered to the ROIs with marginal errors. While inter-regional synchronization with non-zero phase delay has been proposed as a key mechanism in communication between or serial activation of cortical regions, especially during top down controls, no methods have been proposed to determine the stimulation parameters for such stimulations. Furthermore, actual application of the msmp-tACS over bilateral IPS significantly affected the performance, lateralizing the VWM performance towards right visual hemifield trials. While VWM performance was not significantly altered by the stimulation conditions, more right visual hemifield dominance was displayed when the relative performance between right and left visual hemifield trials was assessed. We suggest that the observation is due to the shift in phase relationship between the bilateral IPS affected which information to prioritize between the two hemifields. In the future, bilateral auditory cortices could be stimulated with a phase delay to alleviate symptoms of the schizophrenia patients with auditory hallucinations, and optimal phase delays not investigated in this study could be investigated.

### Supplementary Information

Supplementary Information.

## Data Availability

The data and materials are conditionally available upon request to the corresponding author.
