# Supplementary material for: Transcranial alternating current stimulation over multiple brain areas with non-zero phase delays other than 180 degrees modulates visuospatial working memory performance
Source: Sci Rep. 2023 Aug 5;13:12710. doi: 10.1038/s41598-023-39960-3 (PMC10404219; doi:10.1038/s41598-023-39960-3)
Supplement: Supplementary file 1 — Supplementary Information. [file 41598_2023_39960_MOESM1_ESM.pdf]

Supplementary Materials

Transcranial Alternating Current Stimulation over Multiple Brain Areas with Non-Zero Phase Delays Other Than 180 Degrees Modulates Visuospatial Working Memory Performance

Jimin Park[1*], Sangjun Lee[1‡], Seonghun Park[1+], Chany Lee[2†], Sungshin Kim[3ł] and Chang-Hwan Im[1,4§¶]

[1] Department of Electronic Engineering, Hanyang University, Seoul, Republic of Korea

[2] Cognitive Science Research Group, Korea Brain Research Institute, Daegu, Republic of Korea

[3] Department of Cognitive Sciences, Hanyang University, Seoul, Republic of Korea

[4] Department of Biomedical Engineering, Hanyang University, Seoul, Republic of Korea

1 msmp-tACS: Sham Condition

Eighteen healthy, right visual hemifield dominant volunteers (11 men and 5 women, age: 24.26 ± 2.35) participated for the sham experiment. Since there is no adequate method for analysis of variance comparing the sham group and three repeated measures of one group, we only performed independent t-test. All the p-values are corrected using FDR. Similar to the stimulation group, the LI and K-values for left and right visual hemifield trials were computed.

While we hypothesized that the LI under sham condition would be somewhere in between synchronized (-0.122 ± 0.04) and LR (-0.084 ± 0.04), the mean LI was higher than the LR condition but below 0 (-0.028 ± 0.16). Paired t-test against zero did not show any significance for the sham group (t_{15} = -0.77, p = 0.45). Also, independent t-tests against the three stimulation conditions did not yield significant results (synchronized: t_{15} = 1.74, p = 0.14; RL: t_{15} = -1.73, p = 0.14; RL: t_{15} = 0.97, p = 0.34). The LI distribution for each condition is illustrated in figure S1.

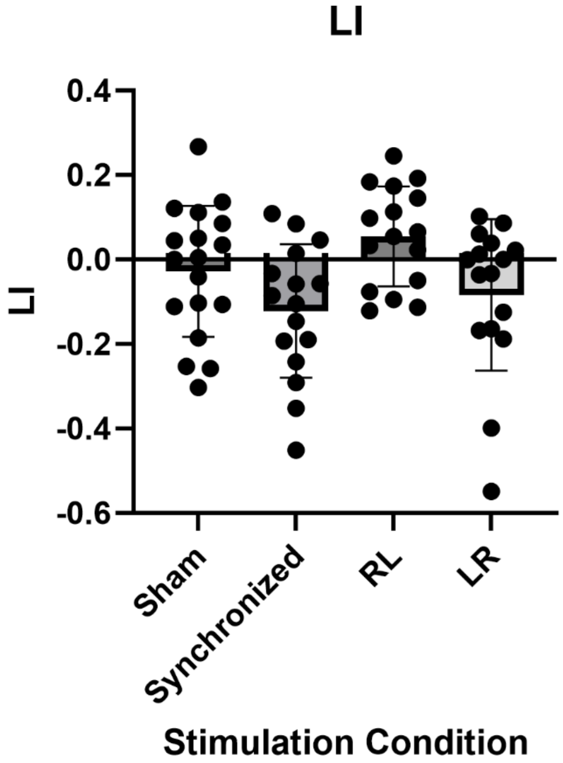

Figure S1. The distribution of LI under sham, synchronized, RL, and LR stimulation conditions.

Interestingly, the K-values, or working memory capacity, was significantly higher compared to the sham group only under the RL stimulation condition. RL is the stimulation condition which the participants exhibited higher LI. The mean K-value was higher under each stimulation condition compared to the sham group (sham: 2.11±0.82; synchronized: 2.38 ± 0.91, $t_{15} = -0.89$, $p = 0.57$; RL: 2.87 ± 0.59, $t_{15} = -3.06$, p = 0.01; LR: 2.21 ± 0.82, $t_{15} = -0.36$, $p = 0.72$). The mean K-values of left hemifield were higher compared to the sham group for all the stimulation condition but was not significantly higher, albeit a trend was observed compared to the synchronized condition (sham: 2.21 ± 0.81; synchronized: 2.86 ± 0.7, $t_{15} = -2.44$, $p = 0.06$; RL: 2.64 ± 0.86, $t_{15} = -1.47$, $p = 0.2$; LR: 2.55 ± 0.68, $t_{15} = -1.31$, $p = 0.2$). The behavioral measures are depicted in figure S2.

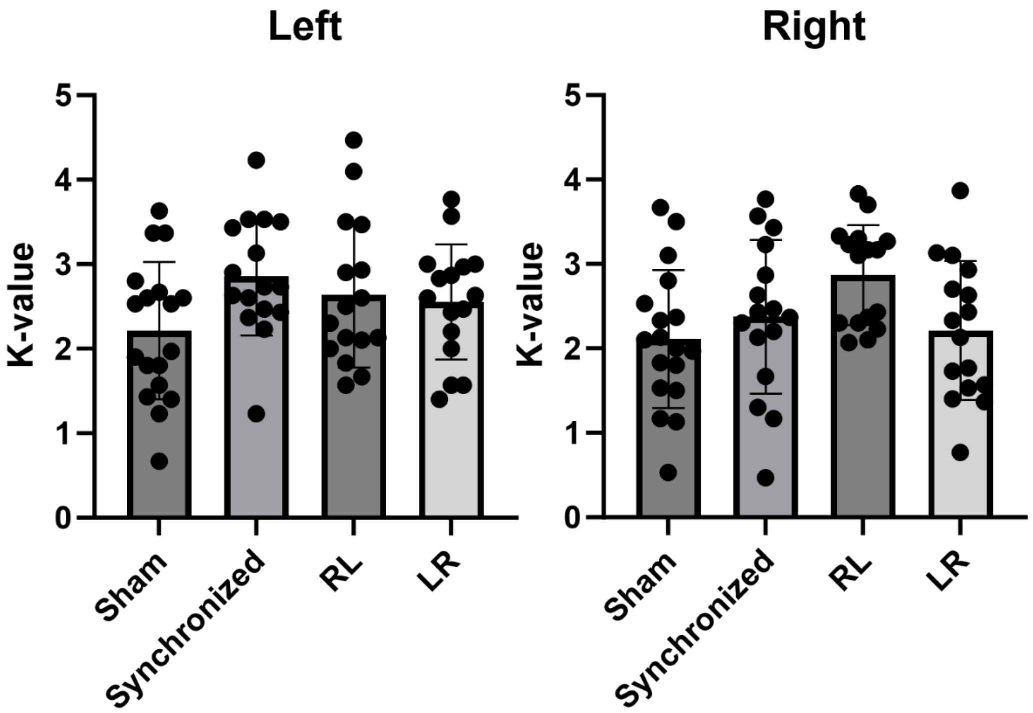

Figure S2. The K-values for left and right hemifield trials under each stimulation condition.

2 Simulation using Colin Model

To validate the feasibility of electrode condition determined by a representative montage, we applied the identical electrode condition over a newly constructed head model. Open MRI dataset, Colin, was used to construct the new head model. The electrode size and height were also the same. We only conducted simulation for RL and LR conditions, since synchronized condition is equivalent to typical tACS. The results showed that the induced phase delays for LR and RL conditions were -97.82° and 86.91° (phase of rIPS subtracted from phase of lIPS), respectively. This is an error of 8.7% and 3.43%, respectively, from desired phase. Also, the E-field amplitude was strong over rPIS and lIPS when simulated in the Colin head model: 0.29 V/m for lIPS and 0.33 V/m for rIPS under LR condition, and 0.29 V/m for lIPS and 0.38 V/m for rIPS under RL condition. The simulation results are illustrated in figure S3.

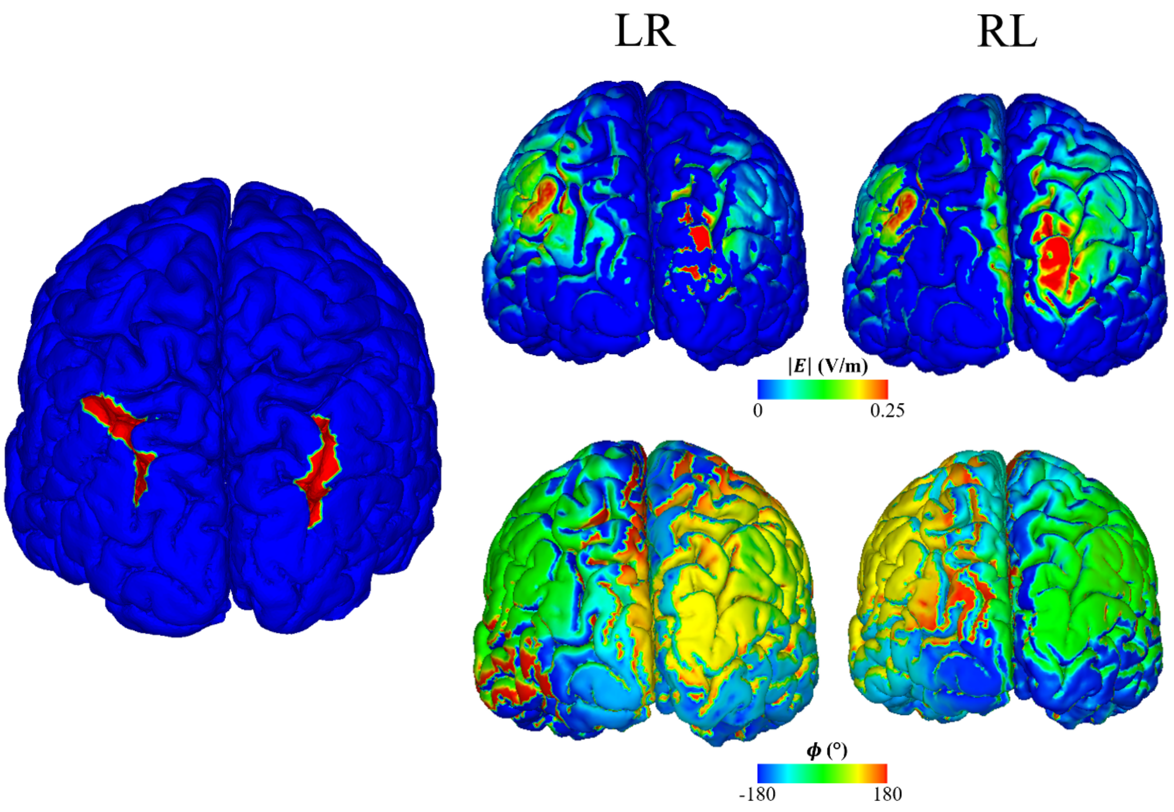

Figure S3. The E-field distribution over the cortex over Colin model when applying the electrode condition used in the case study.
